# The Relationships between Physical Activity, Self-Efficacy, and Quality of Life in People with Multiple Sclerosis

**DOI:** 10.3390/bs9120121

**Published:** 2019-11-21

**Authors:** Marco Guicciardi, Maria Carta, Massimiliano Pau, Eleonora Cocco

**Affiliations:** 1 Department of Education, Psychology, Philosophy, University of Cagliari, 09123 Cagliari, Italy; maria.carta1996@gmail.com; 2 Department of Mechanical, Chemical and Materials Engineering, University of Cagliari, 09123 Cagliari, Italy; massimiliano.pau@dimcm.unica.it; 3 Department of Medical Sciences and Public Health, University of Cagliari, 09042 Monserrato (CA), Italy; ecocco@unica.it

**Keywords:** multiple sclerosis, exercise, efficacy expectations, health behaviors, well-being

## Abstract

Regular physical activity (PA) can enhance the physical and mental health of people with Multiple Sclerosis (MS) because of its impact on muscular strength, mobility, balance, walking, fatigue, pain and health-related quality of life (HRQoL). Previous studies have hypothesized that the relationship between PA and HRQoL is mediated by self-efficacy. The aim of this research is to evaluate whether self-efficacy in goal setting and self-efficacy in the management of symptoms, mediate the relationship between PA and HRQoL, in a similar way to exercise self-efficacy. A sample of 28 participants with MS (18 females) and different levels of physical activity have been recruited and completed the following measures: (a) physical activity (GLTEQ); (b) health-related quality of life (SF-12); (c) self-efficacy in the management of Multiple Sclerosis (SEMS) and, (d) exercise self-efficacy (EXSE). The statistical analysis highlighted that self-efficacy in goal setting mediated the relationship between PA and mental health better than exercise self-efficacy. Our findings suggest that self-efficacy in goal setting can contribute to the adoption and maintenance of regular physical activity for long-lasting times, supporting and increasing the mental quality of life of people suffering from MS.

## 1. Introduction

Multiple Sclerosis (MS) is a chronic, immune-mediated disease of the central nervous system (CNS), with neurodegenerative processes characterised by the loss of the myelin sheath in multiple areas of CNS and consequent formation of scar tissue or sclerosis. 

Different symptoms and dysfunction are associated with MS, such as fatigue, muscle weakness, balance and motor disorders, pain, cognitive impairment, mood disturbances, and depression [1,2,3].

Participation in Physical Activity (PA), particularly exercise training, represents the single most effective non-pharmacological approach for managing symptoms and improving the health-related quality of life (HRQoL) of people with MS [4,5,6]. However, people with MS do less PA compared to non-diseased people [7,8,9], but in similar way as other people suffering from a chronic disease [7]. Thus, according to different studies, people with MS are not getting the minimal amount of daily activity that even sedentary adults without neurologic injury or disease are able to achieve [10,11,12,13]. 

Self-efficacy has been proven to be a key determinant related to adoption and maintenance of physical activity in people with chronic diseases, such as cancer, stroke, spinal cord injury, diabetes, and heart diseases [14,15,16,17,18,19,20]. 

Exercise self-efficacy refers to the confidence people have in their ability to be physically active, such as, for example, their beliefs about achieving 30–150 min of moderate to vigorous physical activity per week [21,22].

This aspect was repeatedly investigated in people with MS by Motl et al. [23,24,25,26,27,28,29], who found (a) a positive relationship between exercise self-efficacy and physical activity; (b) a moderate inverse relationship between MS symptoms and exercise self-efficacy.

The management of MS-related symptoms (e.g., muscle spasms, balance disorders, thermoregulatory difficulties and muscle weakness) has been also invoked to predict both directly, and indirectly through self-efficacy, the adoption and maintenance of PA in individuals with MS [30].

The relevance of symptom’s management in people with MS was also stressed by Bonino et al. [31], who developed a new scale to measure self-efficacy in dealing with physical and psychological problems caused by MS. The management of symptoms (e.g., fatigue or negative emotions) allows the patients to experiment a satisfactory quality of life, which is often reduced in people with MS, even when compared with people suffering from other debilitating conditions [32,33,34].

The goal setting, coupled with self-efficacy, has assumed a pivotal role in intervention studies aiming to enhance physical activity and to improve HRQoL in persons with MS [35]. In order to receive beneficial effects on physical and mental health, physical activity must be carried out regularly. Unlike the exercise training program, which is a specific, structured and supervised form of PA, lifetime PA must be selected by the person, consciously planned and accumulated in short bouts over the day as part of one’s life [36]. However, as Bonino et al. [31,37] highlighted, people with MS are likely to withdraw from planning goals and daily activities, because they perceive themselves as no longer able to do it. Thus, self-efficacy in goal setting should be investigated, along with exercise self-efficacy and self-efficacy in management of symptoms, for their mediation role in the relationship between PA and HRQoL.

It was hypothesized that self-efficacy in goal setting and self-efficacy in management of symptoms mediated the relationship between leisure time PA and mental health, while exercise self-efficacy mediated the relationship between leisure time PA and the physical health of people with MS.

## 2. Materials and Methods

### 2.1. Participants and Procedure

A sample of 28 participants (18 women and 10 men) was recruited from the Regional Reference Centre for Multiple Sclerosis, “Binaghi” Hospital, Cagliari (Italy). The predominance of females in our sample reflected the higher prevalence of MS associated with the individual’s gender. The F:M ratio was estimated within the range 1.9 to 2.7, depending on the geographical latitude [38]. Age range was between 26 and 74 with a mean age of 51.6 years (SD = 14.9); the majority of the participants were married (60.7%), while 32.1% were single; 53.3% were high school graduates, 32.1% had stopped at middle school and 14.3% had completed university. About half of the sample was employed (46.4%) while 39.3% were retired. The sample was characterized by a predominance of individuals with moderate disability (median Expanded Disability Status Scale score 4.2) [39] who were able to walk independently with or without assisting devices. Participants were invited to answer some questionnaires, which took approximately 15 min to complete. Data collection took place on four occasions between 20 September 2018 and 5 October 2018.

### 2.2. Ethics

The study was carried out in compliance with the ethical principles for research involving human subjects expressed in the Declaration of Helsinki and was approved by the Ethics Committee of ATS Sardegna (approval no. 102/2018/CE, 11 September 2018). Written informed consent was obtained from all participants.

### 2.3. Measures

A leaflet composed of a cover page with demographic information (gender, age, marital status, education and employment), the Godin Leisure-Time Exercise Questionnaire (GLTEQ), the Self-Efficacy for Multiple Sclerosis Scale (SEMS), the Exercise Self-Efficacy (EXSE) and the SF-12 Health Survey were administered to all participants.

The Godin Leisure-Time Exercise Questionnaire (GLTEQ) [40] consists of two questions assessing physical activity. The first question asks the participant to write the number of times that he or she has completed at least 15 min of physical activity during the last 7 days. It has three open-ended items that measure the frequency of strenuous (e.g., jogging), moderate (e.g., fast walking), and mild (e.g., easy walking) exercise. Thus, the weekly frequencies of strenuous, moderate, and mild activities are multiplied by 9, 5, and 3 metabolic equivalents, respectively, and summed to form a measure of total leisure activity. The second question asks the participants how many times during the week he or she engages in activities that make them sweat and has three possible answers “always” “sometimes” and “never”. In accordance with different studies (e.g., [23,41]) we chose not to consider this answer in the data analysis because of autonomic nervous system disturbances that result in sweating problems in people with MS. The GLTEQ is a simple, reliable, and valid measure of physical activity that has been widely used with young people in epidemiologic, clinical, and behavioral change studies [23,25,42,43,44,45], concerning person with MS. Since only the first question of the GLTEQ was used, the Cronbach’s alpha was not calculated.

The Self-Efficacy for Multiple Sclerosis Scale (SEMS) [31] assesses self-efficacy related to the management of MS. It comprises 15 items starting from the root statement “*I am confident that I can…*” and is rated on a 5-point Likert scale from 0 (*Not at all confident*) to 4 (*Very confident*). The items provide two sub-scores “Goal setting” (item 2, 3, 4, 5, 6, 7, 8, 13, 14) and “Symptom management” (item 1, 9, 10, 11, 12, 15). According to the authors [37], the scale is characterized by good item functioning, measurement invariance, and good concurrent validity (positive correlations with positive affect, sense of coherence and coping strategies and negative correlations with depression and negative affect). In the current research, the subscales of symptom management and goal setting obtained a good internal consistency (Cronbach’s alpha = 0.83 and 0.86, respectively).

The Exercise Self-Efficacy [46] comprises six items commonly used to assess self-efficacy for physical activity. The participant had to report their level of confidence, ranging from 0% (*not at all confident*) to 100% (*completely confident*), about the sentence “*I am capable of continuing to make a moderate intensity physical activity three times a week for more than 20 minutes, without interruption, for the next week*”. The number of weeks increases by one in every sentence and the last sentence asks the participant how confident he/she is about doing a moderate intensity physical activity three times a week for more than 20 min without interruption, for the next six weeks. In the current research, the scale obtained an excellent internal consistency (Cronbach’s alpha = 0.99).

The SF-12 Health Survey [47] is the short version of the SF-36 already used to assess the quality of life in people with MS [37]. It is a self-report questionnaire composed of 12 items that assess two components: physical health (physical component summary = PCS) and mental health (mental component summary = MCS). Depending on the question, there are different possible answers, for example the first one asks “*In general, would you say that your health is…*” and the answers range from 1 (*Excellent*) to 5 (*Poor*); the second asks “*Does your health now limits you in doing moderate activities?*” and the possible answers are “*Yes, limited a lot*”, “*Yes, limited partially*” and “*No, not limited at all*”; the fourth asks “*Have you accomplished less than you wanted at work and in other activities because of your health status, in the last 4 weeks?”* The answers are “*Yes*” or “*No*”; other items have a range of responses ranging from 1 (*Always*) to 6 (*Never*). The scores were calculated using specific automated algorithms [48]. In the current research, the internal consistency of the scale was found to be good for the global scale (Cronbach’s alpha = 0.83), and for the subscales of physical health (PCS) (Cronbach’s alphas = 0.80) and mental health (MCS) (Cronbach’s alphas = 0.79).

### 2.4. Statistical Analysis

D’Agostino–Pearson tests of normality were performed on all test scores. Spearman rho rank-order correlation were used to examine the associations between variables. Based on the results of correlation analysis, mediation analysis was executed to examine the hypothesized mediation models. Significance was set at *p* < 0.05. Descriptive statistics and Spearman’s rho correlations were calculated using IBM SPSS Statistics version 23.0 (IBM Corp, Armonk, NY). Mediation analysis was carried out by means of the suite Advanced Mediation Models (jAMM), based on R lavaan package [49] and included in Jamovi version 1.0.5 [50].

## 3. Results

Means and standard deviations of the raw item scores of the study variables are shown in Table 1. The GLTEQ scores confirm the low mean level of physical activity of people suffering from MS, coupled with a high heterogeneity of PA behaviors and exercise self-efficacy, testified by standard deviations of both variables.

Spearman’s rho intercorrelations among the studied variables are presented in Table 2. Physical activity, as measured by means of the GLTEQ, was significantly and positively related with exercise self-efficacy (rho = 0.519), but not significantly associated with self-efficacy in symptom management and quality of life; self-efficacy in symptom management was significantly and positively associated with self-efficacy in goal setting (rho = 0.764), exercise self-efficacy (rho = 0.435) and mental component of quality of life (rho = 0.472); self-efficacy in goal setting was positively related with mental component of quality of life (rho = 0.533), as well as with self-efficacy in symptom management.

The correlation analyses revealed that physical component of quality of life (SF-12 PCS) was not significantly associated with physical activity, exercise self-efficacy and self-efficacy for Multiple Sclerosis. Therefore, this variable was not included in the subsequent mediation effect analyses.

**Figure 1:**
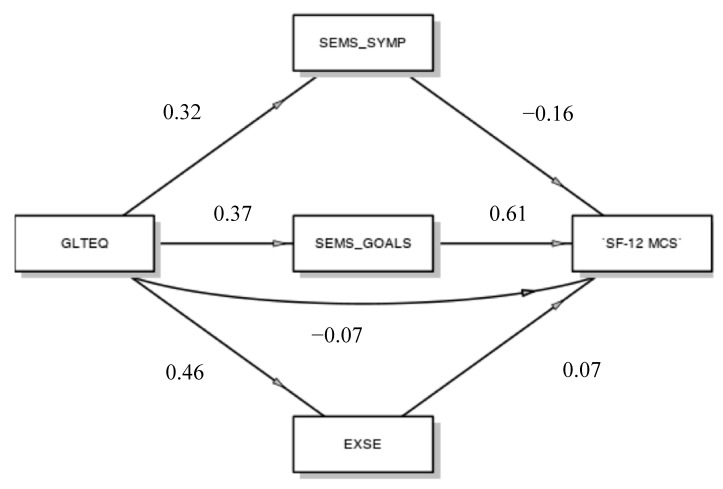
Mediation model. GLTEQ, Godin Leisure-Time Exercise Questionnaire; SEMS_SYMP, Self-Efficacy for Multiple Sclerosis Subscale Symptom Management; SEMS_GOALS, Self-Efficacy for Multiple Sclerosis Subscale Goal setting; EXSE, Exercise Self-Efficacy, SF-12 MCS, SF-12 Health Survey Subscale Mental Component Summary.

Using a generalized linear model, we examined whether self-efficacy in symptom management, self-efficacy in goal setting and exercise self-efficacy all serve as individual mediators in the relationship between PA and mental component of quality of life (Figure 1).

The results indicated that PA did not directly predict mental component of quality of life (β = −0.07), but it did indirectly, through the mediation of self-efficacy in goal setting. Exercise self-efficacy, self-efficacy in goal setting and self-efficacy in symptom management are all appreciably affected by PA (respectively β = 0.46; β = 0.37; β = 0.32), but only self-efficacy in goal setting, which significantly affects the mental component of quality of life (β = 0.61; *p* < 0.001), seems to act as a mediator of the relationship among PA and the mental health of people suffering from MS (β = 0.23; *p* = 0.06).

## 4. Discussion

The aim of the current study was to examine, in people with MS, the relationships between PA and HRQoL, as mediated by different forms of self-efficacy, namely self-efficacy in goal setting, self-efficacy in symptom management, and exercise self-efficacy.

The results of the correlation analyses confirm that different forms of self-efficacy can play a different role on mental and physical components of quality of life. As already reported in previous studies, which considered self-efficacy for control and self-efficacy for functioning [26], self-efficacy for control was positively related with physical and psychological quality of life, while self-efficacy for functioning was significantly correlated with physical quality of life but not with psychological health-related quality of life.

In our study, both self-efficacy in goal setting and self-efficacy in symptom management were positively related with psychological quality of life, while exercise self-efficacy was more related with physical quality of life. Moreover, in accordance with other studies (e.g., [25]), exercise self-efficacy was also associated moderately with PA. As for self-efficacy in MS, namely self-efficacy in goal setting and self-efficacy in symptom management, it showed a more positive relationship with the mental component of quality of life, than with the physical one. Previous studies [31,37] indicated that both forms of self-efficacy in MS correlate with adjustment, but the goal setting dimension has the highest inverse correlation with depression, and a remarkable relationship with sense of coherence, and, above all, with well-being.

People suffering from MS have to overcome more barriers to be physically active: self-efficacy and goal setting can aid them to be more purposeful in planning their activities and in engaging in regular lifestyle PA.

In fact, when considering the indirect relationship between PA and quality of life, as mediated by self-efficacy [27], only mental health appeared affected by self-efficacy and specifically by self-efficacy in goal setting. This result suggests the relevance for MS patients of planning meaningful and realistic goals for their everyday life. This finding is partially consistent with previous literature that states that self-efficacy in goal setting could account for a significant amount of variance in mental health scores of people suffering from MS [37]. Moreover, self-efficacy in goal setting was already correlated with adaptive strategies of problem-solving [31] and the ability to overcome the daily barriers to lifestyle PA was shown to support people with chronic diseases which need a regular and long-lasting commitment [15]. A low adherence rate lifestyle PA is often more related to self-efficacy to cope with the disease than to limitations due to functional incapacity, pain, or other symptoms from which the person suffers [51,52,53,54,55].

The study has some limitations. First of all, it involved a small convenience sample of people suffering from MS, which has limited the possibility of finding stronger and more significant relationships. Secondly, the study was essentially explorative, because little is known about the relationships between PA and quality of life, mediated by specific forms of MS self-efficacy other than exercise self-efficacy. Therefore, the study must be replicated, involving a larger sample, to evaluate if the results observed in this preliminary study are confirmed. Thirdly, the cross-sectional design suggests more caution in the causal interpretation of direct and indirect effects: PA, exercise self-efficacy and self-efficacy in MS are likely to affect physical and mental health-related quality of life; however, it cannot be excluded that the direction of this relationship can be reversed. A longitudinal research design, coupled with more specific statistical procedures, would allow a deeper analysis of the reciprocal relationships among these variables. Fourthly, concerning measurements, it is to notice that many of the participants had difficulties in assessing the level of their leisure PA using the GLTEQ and needed more instructions: for example, some activities used as examples (e.g., ski, snowmobile, hockey), were unusual for our sample, who had difficulty comparing these activities with what they usually do. Although the Godin Leisure-Time Exercise Questionnaire has a long tradition of use in studies on PA conducted with people suffering from MS, we suggest introducing more objective measures of PA, that should be coupled with GLTQ, as recommended for a more structured exercise training intervention [42].

Future studies should take advantage of objective assessment of amount and intensity of PA, which can be carried out on the basis of data collected by wearable devices (e.g., triaxial accelerometers). Such an approach, which is suitable for long-term measurement, has been already successfully tested in individuals with MS for this decade [56].

## 5. Conclusions

The people suffering from MS can benefit from regular PA. However, their rate of PA is lower compared to people suffering from other non-communicable diseases. In addition to the exercise self-efficacy, already investigated in the literature, it seems appropriate to take into account more specific forms of MS self-efficacy. Our results indicate that the self-efficacy in goal setting can mediate the relationship between PA and the mental component of HRQoL, but further studies, conducted on a large scale, are need.

## Figures and Tables

**Table 1:** Descriptive statistics for the variables of interest.

Measure	Mean Score	Standard Deviation	Range of Scores
GLTEQ	17.96	19.11	0–78
SEMS SYMP	24.14	4.66	14–30
SEMS GOALS	33.79	7.04	13–44
EXSE	67.44	33.86	0–100
SF-12 PCS	41.11	11.74	17.35–57.51
SF-12 MCS	53.28	10.35	27.70–70.36

GLTEQ, Godin Leisure-Time Exercise Questionnaire; SEMS SYMP, Self-Efficacy for Multiple Sclerosis Subscale Symptom Management; SEMS GOALS, Self-Efficacy for Multiple Sclerosis Subscale Goal setting; EXSE, Exercise Self-Efficacy, SF-12 PCS, SF-12 Health Survey Subscale Physical Component Summary; SF-12 MCS, SF-12 Health Survey Subscale Mental Component Summary.

**Table 2:** Rank-order correlations among the observed variables (Spearman’s rho).

	2	3	4	5	6
1. GLTEQ	0.259	0.255	0.519 ^**^	0.310	0.010
2. SEMS SYMP		0.764 ^***^	0.435 ^*^	0.191	0.472 ^*^
3. SEMS GOALS			0.338	0.141	0.533 ^**^
4. EXSE				0.315	0.239
5. SF-12 PCS					−0.255
6. SF-12 MCS					

* *p* <0.05, ** *p* <0.01, *** *p* <0.001. GLTEQ, Godin Leisure-Time Exercise Questionnaire; SEMS SYMP, Self-Efficacy for Multiple Sclerosis Subscale Symptom Management; SEMS GOALS, Self-Efficacy for Multiple Sclerosis Subscale Goal setting; EXSE, Exercise Self-Efficacy, SF-12 PCS, SF-12 Health Survey Subscale Physical Component Summary; SF-12 MCS, SF-12 Health Survey Subscale Mental Component Summary.
